# Impact of environmental factors on operative team performance: systematic review and guidance for optimising clinical practice

**DOI:** 10.1007/s00464-025-12362-4

**Published:** 2025-11-11

**Authors:** Connor P. Boyle, James Crichton, Alessandro Sgrò, Sarah H. Michael, Stephen J. Wigmore, Richard J. E. Skipworth, Steven Yule

**Affiliations:** 1 Surgical Sabermetrics Laboratory, Usher Institute, University of Edinburgh, Edinburgh, UK; 2 Clinical Surgery, University of Edinburgh and Royal Infirmary of Edinburgh, Edinburgh, UK; 3 Department of Emergency Medicine, University of Colorado Anschutz Medical Campus, Aurora, CO USA; 4 Centre for Medical Informatics, Usher Institute, University of Edinburgh, 5 Little France Road, Edinburgh, EH16 4UX UK

**Keywords:** Performance, Operating room, Environment, Noise, Temperature

## Abstract

**Objective:**

To evaluate the range and impact of environmental factors on the operative performance of surgical teams.

**Background:**

Optimising surgical performance is critical for patient safety and clinical efficiency. Recent efforts have focussed on both individual (e.g. stress-management, situation awareness) and team (e.g. communication, leadership) factors. However, the influence of environmental conditions within the operating room remains underexplored.

**Methods:**

A systematic review was conducted using EMBASE, Medline, PubMed, and CENTRAL for studies examining the impact of lighting, temperature, humidity, noise, and music on the performance of surgical teams, following PRISMA criteria. Outcomes assessed include operative skills, cognitive workload, and patient outcomes that may be a proxy for operative performance.

**Results:**

Of 9489 articles screened, 34 met inclusion criteria (*n* = 4159 participants). Fourteen studies assessed the impact of noise, eight of music, six of temperature, four of noise and music together, and two of lighting. No studies on humidity met the inclusion criteria. Noise regularly exceeded recommended standards and was associated with increased mental workload and incidence of complications. Although music increased decibels, it was viewed positively by participants, despite not objectively enhancing performance. Increased temperature was associated with less comfort, but not reduced technical or cognitive performance. Increased illumination reduced tiredness in some participants.

**Conclusion:**

Noise consistently exceeds recommended thresholds, is linked to increased cognitive load, and has been associated with complications. In contrast, temperature is individual-specific and may require compromise between team members. There is very limited research on lighting and humidity, and more investigation is warranted before conclusions can be made on these factors. Surgeons should consider modifiable environmental factors as part of a systems approach to optimising operative performance, patient safety, and staff-wellbeing.

**Graphical abstract:**

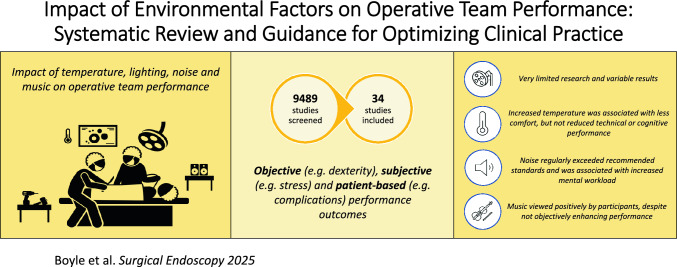

**Supplementary Information:**

The online version contains supplementary material available at 10.1007/s00464-025-12362-4.

For many years, surgical outcomes were thought to be a product of patient factors and the technical skill of the operating surgeons. Recent years have seen greater recognition of the importance of human factors, systems, and non-technical skills (NOTSS), including situational awareness, communication, and organisational culture in surgical performance [1]. However, one system factor that has been neglected in surgery is the physical environment. Exposure to ambient lighting, temperature, humidity, noise, and music are all regulated in non-surgical, high-demand workplaces due to their demonstrated impact on workers’ health, sustained attention, efficiency, and safety. These environmental factors could exert significant influence on operative performance [2] and are not beyond surgeon control, making them potentially modifiable targets for performance improvement.

Lighting is required for optimal vision of the operative field. The lighting directed at the operating table should be at least 10,000 lx, which is the equivalent of clear ambient daylight [3, 4]. Extended exposure to high-intensity lighting can cause surgeon eye fatigue [5], and this negative impact on vision could impair performance. It can also affect the production of melatonin and the sleep–wake cycle, with impaired sleep also potentially impacting performance [6]. Light fittings require repositioning frequently, interrupting the flow of surgery and adding to physical load [7].

Ambient temperature has implications for patient wellbeing and performance of the surgical team. Patient hypothermia is a risk of surgery [2], and there is a well-proven relationship between hypothermia and both intra- and post-operative complications [8]. However, clinical needs have to be balanced with a temperature that is deemed comfortable for the operative team. Although the UK Health & Safety Executive highlights the importance of thermal comfort in the workplace, there is no maximum temperature limit in UK law and there is a reliance on staff and employer consensus [9].

Humidity can vary greatly in the workplace and is a contributory factor to thermal comfort. High humidity environments reduce the capability for heat reduction via sweating, which further limits thermoregulatory processes already hampered by the use of personal protective equipment (PPE), which is commonly worn in the OR environment [9].

Noise is defined as a sound, normally unwanted, that lacks an agreeable quality or is noticeably unpleasant [10]. Operating rooms are environments in which loud noises, including those above regulated levels, can be frequent occurrences [11]. While noise has generally been regarded as a negative influence, the same cannot always be said for music. Some studies have suggested that music in the OR may reduce stress and improve performance [2], and perception is often favourable [12].

Despite the importance of the OR physical environment, the impact of specific elements of it, which are necessary to inform evidence-based strategies, is currently poorly understood. The aims of this systematic review were to examine the variation of five environmental factors (lighting, noise, temperature, humidity, music) in operating rooms and to investigate how they impact operative team performance and patient outcomes.

## Methods

This systematic review was prospectively registered with the PROSPERO database, where the protocol can be accessed (ID: CRD42024588521), and the PRISMA checklist was used when writing the manuscript [13].

### Inclusion criteria

The question posed by this review was “What impact do environmental factors in the operating room (OR) have on the performance of the surgical team?”. Participants in screened studies included staff members working in an OR; surgeons, anaesthetists, and perioperative nurses. Studies that exclusively featured medical students were excluded. The term “intervention” in this review referred to environmental factors in the OR independent of the operating surgeon. These included lighting, temperature, humidity, noise, and music. Surgical gowns and gloves were not included; different sizes, specifications, and brands of gowns/gloves can be worn by different members of the team during the same operation, a difference that is inconsistent with the other included factors.

Only studies that investigated one or more of these environmental factors as a primary aim were included. Simulated studies were included if they met the aforementioned criteria and included a ‘team’ element (for example, a study that included simulated anaesthetic and/or nursing staff in addition to the surgeon). Simulation studies involving a single participant on a bench model were excluded.

Operative performance itself is defined as the process of carrying out an operative procedure to care for a patient, characterised by interdependent skill domains that enable the operative team to accomplish this task; technical, cognitive, interpersonal, and self-regulation [14].

The lack of agreed baseline conditions for lighting, temperature, or music limited the ability to apply a conventional PICO/PECO structure, in terms of defining interventions versus comparators.

### Outcome measures

Three categories of performance outcomes were considered:

- Technical performance: Assessment of technical skills, such as suturing, through any mechanism. Technical skills are defined as goal-directed psychomotor actions during an operation [15].
- Cognitive and psychological load: Stress, mental fatigue, or cognitive workload of the operative team member, assessed through any mechanism. Cognitive load can be defined as the amount of finite working memory resources allocated to meet the cognitive demands of a task
- Clinical outcomes: Patient-related metrics that may serve as a proxy for operative performance in the context of a specific environmental factor, such as post-operative complications.

### Identification of studies

Embase, Medline, PubMed, and Cochrane Central databases were searched from January 1949 to December 2024. A further repeat search was performed in September 2025. There were no language restrictions at the initial stage. The search strategies for the databases (available in supplementary methods) were reviewed and discussed with an expert medical librarian before screening commenced. The reference lists of included studies were checked for additional studies not initially detected, as were prior reviews on individual environmental factors [12, 16].

### Selection of studies

Reference management software (Covidence, Veritas Health Information, Melbourne Australia) was used to import references, remove detected duplicates prior to screening, and then manage the screening process. Abstract screening and the full text review of potentially eligible studies were evaluated independently by two authors (CB and JC). Disagreements regarding eligibility of studies were resolved either in a consensus meeting, or after discussion with a third, senior, author (SY).

### Data extraction and analysis

All data from the included full texts were independently extracted by three authors (CB, JC, and AS) using a pre-defined data extraction template (supplementary methods). This template was reviewed after the initial five data extractions to ensure it was functioning as intended. Data extracted included study details (title, source, lead author, country, year, journal), methods (aim, design, simulation, specialty, multi-centre status), participants (senior staff, trainees, group balance, duration), intervention (environmental factor, measurement, comparison to standards), and outcomes (primary and secondary measures, assessment type, and period). For noise studies, decibel levels were recorded; for music studies, participant approval rates were noted. Key findings on surgical performance were summarised with a one-line conclusion. Data extracted from the included studies are described both narratively and quantitatively in the results section where appropriate. Meta-analysis could not be performed due to the clinical heterogeneity of included studies and the varying methods of data presentation.

### Narrative synthesis

The heterogeneity of included studies led to disparate findings across the range of environmental factors. The findings are initially grouped within the environmental factor studied. For temperature and lighting, the small number of studies means no further sub-division was required. The primary grouping was between study types, namely those that made objective assessments of performance and those that were subjective. If there was an obvious outcome domain that could be grouped, this was also used; for example, the association between noise and complications.

### Quality assessment

Given the range of methods in included studies, the quality assessment with diverse studies (QuADS) appraisal tool was selected to evaluate studies, given its demonstrated strong reliability and ease of use in this kind of systematic review[17] (see Supplementary Fig. S1). Quality assessment was performed independently by three separate reviewers (CB, JC, and AS). Disagreements were settled with consensus.

## Results

**Fig. 1:**
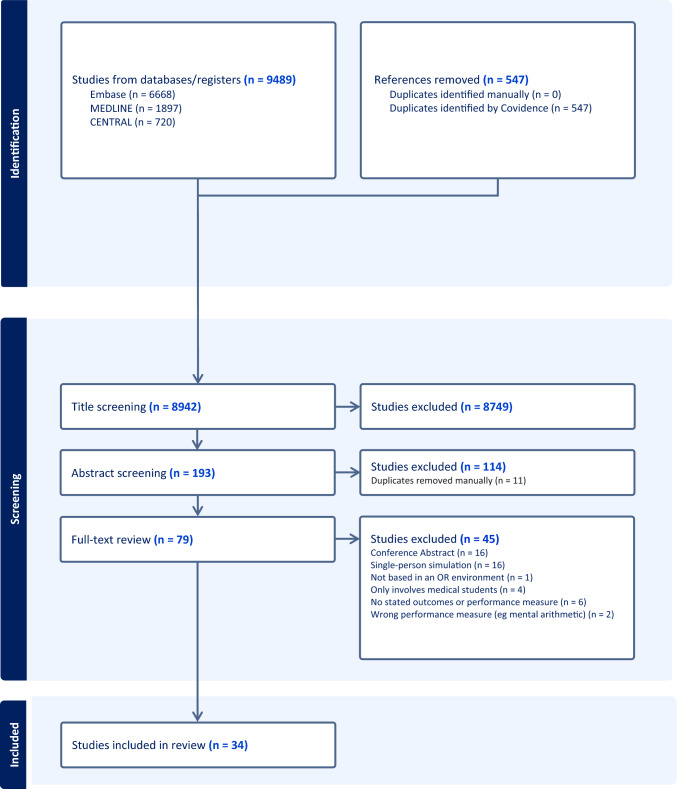
PRISMA: the impact of environmental factors in the operating room on surgical performance

The PRISMA flow-chart is shown in Fig. 1. From an initial 9489 articles, 34 studies met the criteria for data extraction. A one-sentence summary of each paper, produced during the extraction process, has been included as a supplementary material (S3). No studies on humidity met the inclusion criteria.

### General study and population characteristics

The 34 studies included at least 4159 participants (2 studies did not state participant sample size) and 2635 surgical cases (9 studies did not state case sample size). General study and population characteristics are shown in Table 1.

**Table 1:** General study and population characteristics

Author (Year)	Country of study	Study design	Environmental factor	Participants	Population	Specialty	Cases
Arabaci (2021)	Turkey	Cross-sectional observational	Noise	50	Surgeons, anaesthetists, nursing staff	General Surgery, Orthopaedics, Urology	403
Brommelsiek (2022)	USA	Cross-sectional observational	Noise	60	Surgeons, anaesthetists, nursing staff, students	Multiple (4 or more specialties)	59
Byrne (2023)	USA	Randomised crossover trial	Temperature	30	Surgeons	Orthopaedics	120
Chandrasekar (2024)	India	Non-randomised experimental	Music	3	Surgeons	Neurosurgery	16
Cheriyan (2016)	USA	Non-randomised experimental	Noise	4	Surgeons, anaesthetists, nursing staff	Urology	3
Dholakia (2015)	UK	Cross-sectional observational	Noise	2	Surgeons	General Surgery	64
Engelmann (2014)	USA	Non-randomised experimental	Noise	16	Surgeons, Anaesthetists, Nursing Staff	Paediatric	144
George (2011)	India	Cross-sectional descriptive	Music	100	Surgeons, Anaesthetists, Nursing Staff	Not stated	Not stated
Gülsen (2021)	Australia	Cross-sectional observational	Noise	92	Surgeons, anaesthetists, nursing staff, radiology techs	Multiple	Not stated
Han (2022)	China	Randomised crossover trial	Noise, Music	114	Surgeons	General Surgery	57
Hemphala (2020)	Sweden	Non-randomised experimental	Lighting	114	Surgeons, anaesthetists, nursing staff	Not stated	
Hogan (2015)	USA	Non-randomised experimental	Noise	Not stated	Surgeons, anaesthetists, nursing staff	Multiple	118
Idrees (2024)	India	Cross-sectional observational	Noise	6	Surgeons	ENT	37
Kurmann (2011)	Switzerland	Cross-sectional observational	Noise	Not stated	Surgeons, Nursing Staff	General Surgery	35
Lehrke (2022)	Germany	Non-randomised experimental	Noise	81	Surgeons, anaesthetists, nursing staff, perfusionists	Urology and Cardiothoracics	53
Makama (2010)	Nigeria	Cross-sectional descriptive	Music	162	Surgeons, anaesthetists, nursing staff	Multiple	Not stated
Morghen (2009)	Italy	Cross-sectional descriptive	Lighting	134	Anaesthetists, Nursing Staff	Multiple	134
Narayanan (2024)	New Zealand	Cross-sectional descriptive	Music	120	Surgeons	General Surgery, Vascular, Paediatric	Not stated
Narayanan (2023)	South Africa	Cross-sectional descriptive	Music	107	Surgeons	Multiple	Not stated
Narayanan (2018)	New Zealand	Cross-sectional descriptive	Music	101	Surgeons, anaesthetists, nursing staff, anaesthetic technicians	Not stated	Not stated
Padmakumar (2016)	UK	Cross-sectional descriptive	Noise, Music	410	Surgeons, anaesthetists, nursing staff, ODPs, HCAs	Multiple	Not stated
Palejwala (2023)	Australia	Non-randomised experimental	Temperature	10	Surgeons	Plastics	40
Palejwala (2019)	Australia	Non-randomised crossover trial	Temperature	17	Surgeons, anaesthetists, nursing staff, PT, OT, anaesthetic technicians	Plastics	34
Palejwala (2024)	Australia	Cross-sectional descriptive	Temperature	33	Surgeons	Plastics	Not stated
Peisl (2024)	Switzerland	Non-randomised experimental	Noise	1165	Surgeons, Anaesthetists, Nursing Staff	General Surgery, Cardiothoracics	294
Shover (2021)	USA	Non-randomised, blinded crossover trial	Music	19	Surgeons	General Surgery, Vascular	57
Srivastava (2021)	India	Cross-sectional descriptive	Noise, Music	290	Surgeons, Anaesthetists	Multiple	Not stated
Stadler (2023)	Austria	Non-randomised experimental	Noise	5	Surgeons, Anaesthetists, nursing staff	Orthopaedics	10
Tseng (2022)	Taiwan	Non-randomised experimental	Noise, Music	36	Nursing staff	General Surgery, Orthopaedics	70
Tsiou (2008)	Greece	Cross-sectional observational	Noise	684	Surgeons, anaesthetists, nursing staff	Multiple	43
Ukegjini (2020)	Austria / Switzerland	Non-randomised experimental	Noise	50	Surgeons	General Surgery	664
Ward (2021)	Australia	Non-randomised crossover trial	Temperature	17	Surgeons, Anaesthetists, Nursing Staff, PT/OT	Plastics	34

### Temperature

Six studies assessed the impact of temperature on operative team performance, incorporating 115 participants and 268 surgical cases [18–23]. The only surgical specialties assessed were Plastics and Orthopaedics. Four of the studies assessed the impact of an increase in OR temperature on aspects of operative performance, including cognition, manual dexterity, and perception [20–23]. Manual dexterity, assessed using the Purdue Pegboard method [24], did not deteriorate in any of the studies with an increase in OR temperature. One study [22], a non-randomised crossover trial, demonstrated decreased response times and impaired cognitive accuracy with increased temperature, but a subsequent study from the same team [20] showed no impact on objective cognitive performance with higher temperatures despite participants’ subjective perception of performance impairment.

The impact of cooling vests to improve thermal comfort and preserve cognitive function was assessed in a randomised crossover trial [18]. While thermal comfort and surgeon comfort were both significantly increased by the use of the vest, there was no discernible benefit on cognitive function.

### Lighting

The literature search revealed only 2 studies that assessed the impact of OR lighting on operative team performance [4, 25]. The first study focussed on anaesthetists and nursing staff in ORs and intensive care units and did not include surgeons [25]. One hundred and thirty four participants from 3 different hospitals were surveyed about their perceptions of OR lighting, the results of which were correlated with objective lux measurements. The study found that an increase in illumination did not have a significant effect on staff stress or perceived performance. The second study found the opposite [4]; it demonstrated an increase in general illuminance in the OR improved visual ability for staff, with a concurrent decrease in subjective reported tiredness. All of the cases in this study were performed during daylight hours and did not include those working an evening or night shift.

### Noise

Fourteen studies assessed noise and operative team performance [26–39]. They are summarised in Table 2. The mean noise level in decibels recorded in operating rooms across the studies was 56.8 dB with a mean maximum level of 89.3 dB.

**Table 2:** Studies assessing noise and operative team performance

Outcome Category	Author (Year)	Subjects	Primary outcome measure	Mean and/or max decibel level?	Key findings for operative performance
Post-operative outcomes or complications	Dholakia (2015)	2	Association of noise level during operation and subsequent surgical site infection	Mean pre-op = 47.6, mean in SSI group = 59	Noise greater in SSI group, from 50 min onwards (mean increase 11.337 dB). Additional hospital cost for SSI patients was £243 per patient
	Engelmann (2014)	16	If a noise-reduction programme in an operating theatre reduces surgical complications	Mean = 63, multiple peaks > 80	Intervention associated with reduction in peak/mean noise levels and a reduction in complications, but no significant difference in biometric measures of surgeon stress
	Kurmann (2011)	Not stated	SSI rate 30 days after surgery	Mean = 25.2	Significant association between sound level in OR and development of SSI. Intraoperative noise volume is a possible marker of difficult operation, but study did not demonstrate association between the duration of operation and intraoperative noise
	Peisl (2024)	1165	If higher noise levels corresponded to an increase in surgical complications	Mean = 53–63	Noise levels higher during operations with postop complications. Operations with higher objective difficulty showed significantly higher noise levels and were more likely to lead to postop complications. However, objective surgical difficulty was sole significant predictor of postop complications and noise does not independently predict postop complications
	Ukegjini (2020)	50	Would a visual noise meter decrease noise levels in the OR and subsequently decrease postop morbidity	Mean = 53.8, Max = 76.3	Average noise level significantly decreased by 3.8 dB (A) after intervention with the feedback device. Average noise levels were statistically significantly greater in short procedures of less than 90 min and in easy operative procedures. Mean noise level in the OR had no significant impact on either the frequency of severe complications or the 90-day mortality
Noise reduction and performance	Hogan (2015)	Not stated	Implementing noise reduction strategies in the OR	Mean = 62, Max = 83	An educational programme focussed on noise reduction strategies was successful at significantly reducing mean and maximum noise levels in the OR
	Lehrke (2022)	81	Impact of the use of a noise reduction system (SOTOS) on subjective stress and exhaustion as reported by theatre staff	Mean = 62.75–65.36	Subjective staff opinion found that, in the group that used the SOTOS system, exhaustion and stress were not as pronounced as in the control group, and there may be a positive effect. There was a lot of variability in the results, and clinical/statistical significance is questionable
	Stadler (2023)	5	Impact of noise-cancelling headphones (ANCH) in improving communication, mental load and performance during TKAs	Mean = 61, Max = 93.5	No perceived benefit for surgeons or scrub nurses during TKA in overall communication, performance, teamwork, and mental load. Only anaesthetists seemed to benefit
Impact on subjective performance	Arabaci (2021)	50	Does noise level in the OR affect stress or workload of OR staff?	Mean = 63.67, Max = 91.9	Weak correlation between noise level and stress and workload scores amongst staff. Average and maximum noise levels in theatre dramatically exceed WHO safe limits
	Brommelsiek (2022)	60	Which noises in the OR were loud/distracting, which ones interfered most with team communication and job performance	Mean = 60, Max = 105	93% answered yes to the question "noise in the OR intereferes with your ability to understand your colleagues" Multiple ongoing conversations among team members were the most interfering with crucial team communication
	Cheriyan (2016)	4	Impact of noise level during a simulated PCNL on staff communication, and ability to hear words spoken by the surgeon	Mean = 53.49 ambient and 81.78 with music + equipment	Communication is impaired when the PCNL machine and music are turned on, particularly the anaesthetist and nurse positions. Total PCNL noise + music was louder than a car or pickup truck at 30 feet, or a freight train at 45mph
	Gülsen (2021)	92	Noise levels in the OR	Mean = 60.9, Max = 86	82.6% experienced both physiological and psychological negative effects of noise in the OR
	Idrees (2024)	6	Relationship of noise levels in theatre to surgeon’s salivary cortisol, representing surgeon stress	Mean = 70, Max = 90.06	Surgeon was more significantly affected by noise, especially during critical phases. Difference in baseline cortisol and post-noise exposure cortisol levels of surgeon was significant. Maximum and mean noise levels were significantly associated with post-noise exposure salivary cortisol elevation in the surgeon
	Tsiou (2008)	684	To measure noise pollution in operating rooms and identify its sources	Max = 106	Noise often in excess of 60 dB (A), with the suction apparatus often > 100. Anaesthetists and nursing staff reported greater sensitivity to noise than surgeons. Surgeons were most affected by external noise and conversation, whereas rest of the team reported equipment/aircon was bigger issue

There were some notable results. Two studies focussed on the relationship between noise and surgical site infection (SSI) [28, 33]. Over 99 total cases, there was an association between intraoperative noise level and an increased risk of an SSI. There was an increase above the baseline noise level in the SSI groups of 10.1 dB (*p* = 0.001) and 11.3 dB, respectively. Noise also increased as the duration of the operation increased, particularly from 50 min onwards. This finding was replicated in a recent study that investigated the rate of overall complications [35]. There was a significant increase in noise in the final 10% of the operation and, again, there was a higher noise level in operations that had post-operative complications. However, the difference between the two groups was lower at 0.28 dB (A), and noise was not shown to be an independent predictor of complications; rather, it demonstrated a difficult operation, with operations with higher objective difficulty significantly associated with higher noise levels.

Three studies investigated noise reduction interventions and subjective performance measures. Two of these utilised noise-reducing headphones. Although one study [34] found that stress and exhaustion were not as pronounced in the group using a sound reduction system, the clinical significance was felt to be questionable. The second study [36] found no perceived subjective benefit of noise-reducing headphones on performance, stress, and also noted a negative impact on communication. The third study [38] used a visible noise meter in the operating room. This did reduce perceived surgeon stress and the overall noise level by 3.8 dB (*p* < 0.001) but had no impact on post-operative morbidity.

Remaining studies focussed on the impact of noise on subjective performance. 85% of the 836 surveyed OR staff felt that noise negatively impacted communication and performance [26, 30, 37]. Novel metrics have been utilised, such as the relationship between surgeon salivary cortisol level, surgeon stress, and noise in the OR [32]. There was a significant positive relationship between surgeon stress experienced at different points of an operation and the level of salivary cortisol. The post-noise-exposure salivary cortisol levels were also significantly higher than baseline levels, implying a direct effect of loud noise on the autonomic and endocrine systems of the participants.

### Music

Seven studies, with 631 participants, looked at the impact of music on operative performance [40–46]. These results are summarised in Table 3.

**Table 3:** Studies assessing music and operative team performance

Author (Year)	Subjects	Population	Surgical specialty	Primary outcome measure	% in favour of OR music?	Key findings for operative performance
Han (2022)	114	Surgeons	Gen Surg	Whether music (compared to general ambient noise) would improve performance in a laparoscopic task	n/a	Teams performing with music, rather than the equivalent level of noise, performed better at the task (both quicker and with less mistakes)
Padmakumar (2016)	410	Surgeons, anaesthetists, nursing staff, ODPs, HCAs	Multiple	Which human factors in theatre were adversely affected by noise	78%	80% of respondents felt communication was adversely affected, 77% concentration, 61% stress level and 50% performance in general. Most respondents (*n* = 385, 78%) thought that music did not have an adverse influence on them
Srivastava (2021)	290	Surgeons, Anaesthetists	Multiple	Staff opinion on the impact of noise on communication and stress in the OR	72.90%	Majority (86%) felt background noise in OR negatively affected working, particularly communication. Most respondents did not feel music had an adverse impact (72.9%). Surgical suction biggest source of noise pollution
Tseng (2022)	36	Nursing staff	Gen Surg, Ortho	Impact of different sound conditions on performance of nurses in the OR	n/a	Noise increased mental workload and anxiety but no impact on situational awareness. High volume music (similarly to noise) increased mental workload and anxiety
Chandrasekar (2024)	26 in survey, 3 in experimental study	Neurosurgeons	Neurosurgery	Performance on 3 neurocognitive tasks having listened to music for 20 min prior, versus no music prior	n/a	No statistically significant improvement in cognition, although general trend was towards better performance in the music group. Music appeared to alleviate subjective stress
Makama (2010)	162	Surgeons, anaesthetists, nursing staff	Multiple	Is the presence of music in the OR viewed positively by staff	89.50%	The vast majority of participants preferred to have music in the OR. Perceived to increase performance and other beneficial effects on anxiety and stress
Narayanan (2024)	120	Surgeons	Gen Surg, Vascular, Paeds	Staff perception of the effect of music on stress/mental workload in the OR	76%	Majority felt music improved temperament (68%), stress (59%), how mentally fatiguing a procedure felt (67%) and anxiety (56%). Opinions on communication were divided and a notable minority do find it distracting
Narayanan (2023)	107	Surgeons	Multiple	Surgeons’ perceptions of the effect of music on their own experience of stress and mental workload	74%	Music widely viewed as a positive. Respondents largely felt music improved their own surgical performance. A sizeable minority (27%) reported communication worsened with music
Narayanan (2018)	101	Surgeons, anaesthetists, nursing staff, anaesthetic technicians	Not stated	Staff perception of the impact of music in the OR	70%	Music was seen as improving calmness, mood and overall performance (all *p* < 0.001). However, majority thought that communication was worsened and that it was negative in a crisis
Shover (2021)	19	Surgeons	Gen Surg, Vascular	Impact of music on performance of a simulated surgical task	89.00%	Relaxing music did not improve surgical performance in junior surgical trainees

Two experimental studies looked at music and objective performance. A blinded crossover trial [40] found that, although 89% of the trainee participants viewed music in the OR positively, it did not objectively improve surgical performance. Another study [42] had similar findings; although the surgeons involved felt that music improved their perceived stress, there was no significant improvement in either cognition or overall performance if it was played intra-operatively.

Four studies included noise and music together. A randomised crossover trial [47] involving 114 participants found that teams performing laparoscopic surgery completed the simulated tasks more quickly and with fewer mistakes if music was playing, rather than with an equivalent level of background noise. This impact was more pronounced with less experienced surgeons. Two of the studies were cross-sectional descriptive studies, utilising surveys that included a combined 850 participants [48, 49]. A mean of 83% and 83.8% of participants in each of the two studies felt noise negatively impacted on communication in the operating room, with 50% in one study feeling it had a negative impact on performance in general [48]. The subjective opinion on music was very different; a mean of 78% and 73% of participants in the two studies, respectively, felt music did not adversely affect their perceived operative performance (Table 4).

**Table 4:** Summary table

Environmental factor	Number of studies	Participant number	Outcome types	Important findings
Noise	14	2215	Association with post-operative complications Impact of noise reduction on performance Impact on subjective staff performance	The majority of OR staff felt it negatively impacted performance. It has been linked objectively to an increase in complications (such as SSIs), but there is not yet evidence for an effective, scalable noise-reduction solution
Music	11	1481	Task completion with music versus no music Impact on objective performance Impact on subjective stress, communication	While not objectively improving performance, it is viewed favourably by the majority of team members
Temperature	6	115	Impact of increased temperature on cognition and manual dexterity Impact of cooling vests on performance	Increased OR temperature does not affect objective dexterity or cognitive function in isolation, but can exacerbate subjective stress
Lighting	2	248	Association between increased illumination and subjective stress/fatigue	Evidence too limited to draw significant conclusions
Humidity	0	0	n/a	n/a

### Quality assessment of studies

The mean QuADS score of the included studies was 24 out of 39, with a median score of 23. The median QuADS scores for the different environmental factors were as follows: 20 for music, 20 for music and noise, 24 for noise, 30 for temperature, and 27 for lighting. A full breakdown is included in the supplementary material (S2). A summary of the quality assessment of studies is demonstrated in Table 5.

**Table 5:** Quality assessment of the included studies

Environmental factor	Number of studies	Types of study	Median QuADS score (maximum of 39)
Noise	14	Experimental (7), cross-sectional observational (7)	24
Music	12	Cross-sectional descriptive/survey based (8), experimental (2), randomised crossover trial (1), non-randomised crossover trial (1)	20
Temperature	6	Non-randomised crossover trial (3), randomised crossover trial (1), experimental (1), cross-sectional descriptive/survey based (1)	30
Lighting	2	Cross-sectional observational (1), cross-sectional descriptive (1)	27
Humidity	0	n/a	n/a

This pattern reflects the study types in each subgroup; the survey-based cross-sectional studies were primarily in the music and noise categories, compared to objective, experimental studies in the lighting and temperature groups. The overall median score of 23 indicates that, in general, studies in this field have not tended to be of significantly high quality. Even when separated out into subgroups, none of the median scores were above 30 (out of a total of 39).

## Discussion

This systematic review analysed the relationship between environmental factors in the operating room and performance of the operative team. Although none of these factors are new, and indeed have been present for decades within that environment, all of the included 34 studies have been published since 2008.

**Fig. 2:**
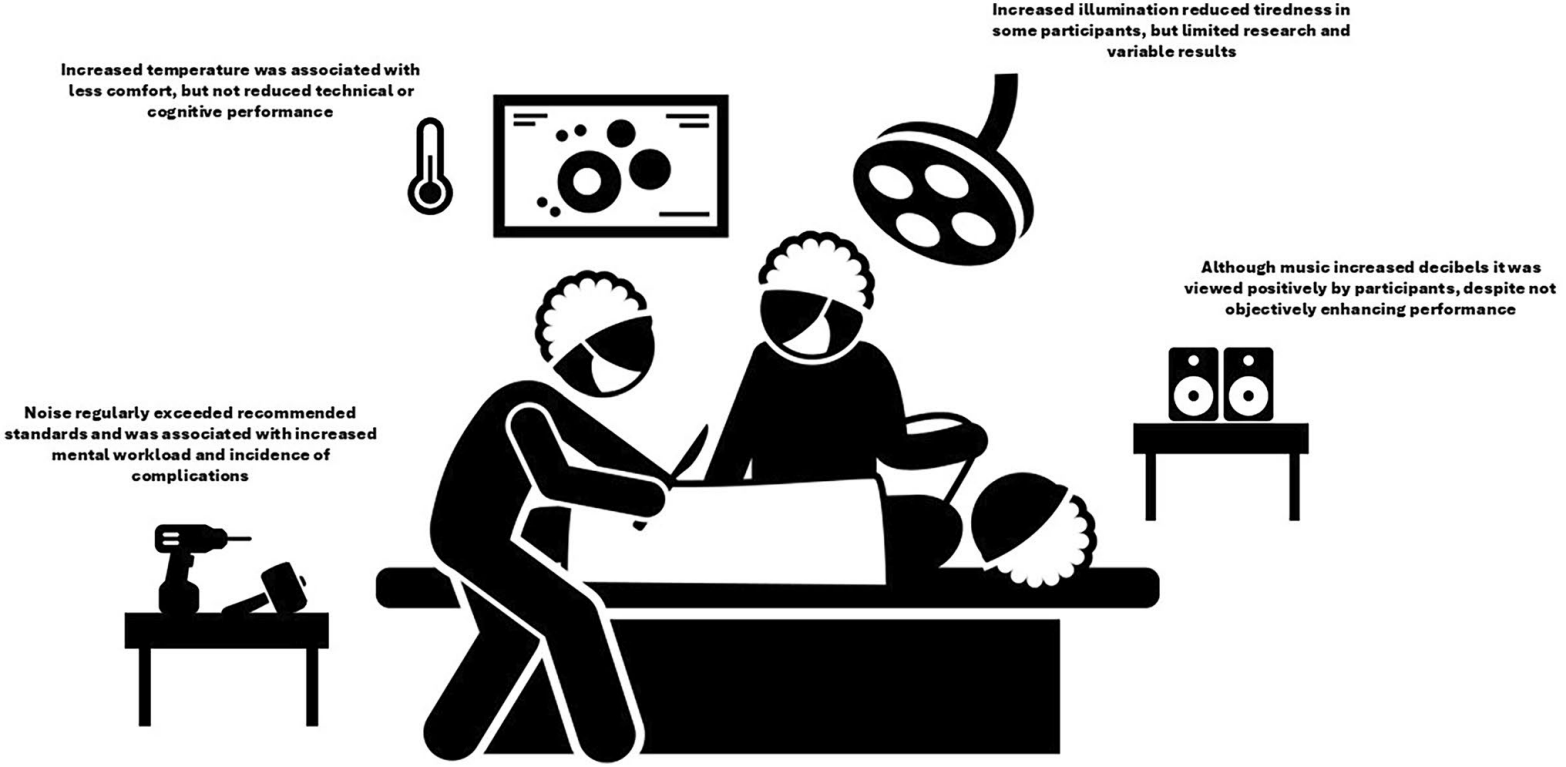
Impact of the physical environment in the operating room

There are several key findings (Fig. 2). Noise levels in operating rooms are routinely high and certainly higher than recommended safety levels. Excessively high noise can increase cognitive workload, which in turn could negatively impact team performance and surgical outcomes. More studies are needed, however, to determine whether this is causal or associated. The impact of music on performance is context-dependent; although most staff view it positively, there is a concern that it could negatively affect communication in a crisis situation. High temperatures lead to obvious staff discomfort and may have some impact on cognitive function, but there is no clear consensus on this.

There is very limited literature on lighting, despite its critical importance in the OR. Whether this reflects adequate current design or an area of oversight is open to question. The first study that investigated OR lighting and performance focussed on the anaesthetic and nursing teams, rather than the surgeons [25]. This looked at the relationship between the actual degree of illumination (in lux) and perceived performance, with the hypothesis that increased lux would improve cognition and subjective mental performance [25]. The nature of illumination, particularly blue light, and human cognition has become increasingly topical. The European Commission’s scientific committee has released an extensive report on the health effects of artificial light [50], and there is concern that it can negatively impact human cognition [51]. While this study did not find decreased illumination resulted in decreased mental performance, there was no control comparator.

The second study focussed on surgeons and nursing staff [4] and highlighted the opposite. Not only did increased illumination in the OR improve visual acuity, it also reduced perceived tiredness among staff. Given there are only two studies on lighting and the results are contrasting, no firm conclusions can be made, and further research is required; for example, a simulated study comparing different luminance levels on operative performance. Furthermore, given the impact of artificial light on melatonin production [51], there may be additional differences specific to night-shift workers that have not been investigated. The long-term impact of blue light on staff performance and wellbeing is outside the scope of this review.

The impact of altering the thermodynamics in the OR does not appear to affect technical components of performance such as manual dexterity. Even when participants (a broad spectrum of roles, including surgeons) felt that their performance was impaired at higher temperatures, objective testing found no obvious decline in cognitive or technical function. Clearly, thermal comfort of the staff is important, and high temperatures should not be tolerated if there is no clear reason to do so. However, optimal temperature of the patient is of primary concern to prevent hypothermia and negative outcomes [8], with patients often actively warmed. The comfort of the operative team is secondary to this. Established gender discordance with workplace temperature and overcooling [52] shows that simply lowering the OR temperature is not a viable solution, and this needs to be judged on a case-by-case basis with the rest of the team. The differences in team role, gender, patient, and the operation itself reflect the relative complexity of thermodynamic optimization.

While defined and viewed differently, when measured in decibels, noise and music ultimately cause the same effect on the OR environment. What is interesting is the difference in how they are perceived by members of the team. The Control of Noise at Work regulations came into force for industry in the UK in 2005 [53] and advises that employers must offer information and training at 80 decibels (dB) and hearing protection above 85 dB. The mean maximum noise level across the included studies was above 85 dB, the level at which regulated standards in the UK advise hearing protection be provided by employers [53]. In several studies in the review, particularly in orthopaedic surgery, the noise level exceeded 100 dB. This is the equivalent noise level a person would experience operating a chainsaw [54], and it is unsurprising that it has both a negative impact on operative performance and perception from staff members.

Music, however, is viewed positively by the vast majority of staff. Although there is no objective performance benefit, they feel it has no negative impact and that it is psychologically beneficial for stress. On a longer-term basis, the use of music to reduce stress could potentially translate to improved performance.

Another important nuance is the relationship between noise and complications. A recent study [35] highlighted noise was not an independent predictor of complications, rather a marker of operative difficulty. In practice, this may reflect that challenging cases require more discussion between members of the team, potentially in an urgent fashion that would necessitate a raised tone of voice. For example, in a patient with significant bleeding, the surgeon must clearly alert anaesthetist, who then requests blood for transfusion, asks for extra equipment, and calls for help. Each step amplifies the noise level because the cascading problem demands louder, faster, concurrent communication. It is an important distinction and highlights the need for contextual assessment of the operative situation, rather than simply the decibel level.

Reducing the negative impact of noise is a challenge. The studies assessing noise reduction technology [34, 36] did not demonstrate a clear benefit. Displaying the real-time noise measurements did lead to a quieter operating environment [38], and although that particular study was assessing post-operative morbidity as an outcome, it is known from the other included studies that reducing the noise would at least subjectively improve communication and performance. Routine measurement and display of noise levels, especially in departments like Orthopaedics, could allow a more thorough occupational assessment and provide evidence to hospital management that training is recommended. Visual sound display meters have been used in intensive care and neonatal settings to good effect [55, 56], and if behavioural change leads to an improvement in how the surgical team feel they communicate, that can only be viewed as a positive.

This review identified studies with both objective (e.g. dexterity, surgical complications) and subjective (e.g. stress, perceived performance) outcomes. Studies with objective outcomes had higher QuADS scores and would be viewed as scientifically more robust in comparison to survey-based, subjective-outcome studies. Paradoxically, although objective measures may provide stronger evidence, they can be harder to implement in clinical practice. For example, preferred operating room temperature varies by individual, so no objective metric will suit everyone, whereas a large majority of team members subjectively advocating for the use of music in the OR may offer an easier, more feasible pathway for implementation.

### Implications for future research

There are clear research gaps in this topic. We suggest the following recommendations as a call to action, both to aid the implementation of the review findings and in the planning of future research:

**Table:** 

Recommendations
Further research and live trials of visible noise meters in the operating room
Occupational health advice in ORs with consistently high noise levels
A pragmatic approach to music; while its use is likely beneficial, a plan is required for quickly silencing it in a crisis situation
Discussion of the OR temperature as part of the pre-operative checklist
Regular objective assessment of OR lighting to ensure it is still at recommended levels, rather than the assumption that this is the case, and well-designed studies to assess this

### Limitations

This was a broad review, and as such study heterogeneity is high. It should be acknowledged that, although the overwhelming majority of the survey-based studies found that music was viewed positively, variability in survey methodology and the absence of standardised questions limit direct comparability. Some of the studies did not specify the number of participants or their seniority, which reduced our ability to assess variations by experience level.

The relatively few studies on lighting and temperature mean that findings for these factors are less conclusive than those for noise and music. This is also the case when considering surgical specialty; for example, all studies on temperature were conducted within Plastic and Orthopaedic surgery. This raises the question as to whether the findings of those studies would be transferable to General or Cardiac surgery without further specific research.

All of the median QuADS scores in the quality assessment were relatively low (with no individual factor having a score of more than 30, out of a total of 39). This is particularly evident for studies on noise and music. The disparate quality of evidence limits our ability to state firm conclusions or recommend practice changes; however, several important gaps in required evidence were identified for future study.

The use of the QuADS score itself can be viewed as a limitation; in some circumstances, a GRADE assessment may have been a preferred alternative. However, QuADS has considerable validity evidence for assessment of mixed-methods studies, and we felt that for clarity and integrative understanding of study findings, that using the same tool to evaluate every included study was preferable.

Two authorship groups have more than one paper included in this review. This is particularly relevant for the sub-grouping of temperature, where five of the six studies were conducted by the same group between 2019 and 2025. Discussion of the findings from the temperature studies is, essentially, a discussion of this team’s research and introduces a risk of team bias. However, it is important to emphasise that each study was reviewed individually, by multiple reviewers, each of whom conducted the extraction process exactly as with the other included studies. The extracted data were unique to each study, as each had independent research questions, and they were not analysed as a grouping.

Finally, while this review prioritised ‘team-based’ studies to assess operating room performance, excluding single surgeon simulated studies may have limited insights into the individual impact of music on performance.

## Conclusion

The impact of environmental factors on operative performance is wide-ranging and variable between staff groups, locations, and the factor itself in question. In isolation, the benefits of altering an individual factor may be minor or subjective; however, as with elite sport, the scope for ‘marginal gains’ in performance is highly relevant to surgery. Improving individual aspects of the operative environment, in combination, may have the potential to lead to substantial performance benefits [57, 58]. The physical OR environment is not beyond control or improvement, and recognising when it is sub-optimal and impacting on performance is another important facet to the human factors approach in surgery.

## Supplementary Information

Below is the link to the electronic supplementary material.

Supplementary file1 (DOCX 21 KB)
