# Supplementary material for: Impact of environmental factors on operative team performance: systematic review and guidance for optimising clinical practice
Source: Surg Endosc. 2025 Nov 11;39(12):7983–99. doi: 10.1007/s00464-025-12362-4 (PMC12708707; doi:10.1007/s00464-025-12362-4)
Supplement: Supplementary file 1 — Supplementary file1 (DOCX 21 KB) [file 464_2025_12362_MOESM1_ESM.docx]

**Supplementary Materials - Index**

| Supplementary Methods |  |
| --- | --- |
| Search Criteria | Pages 2-4 |
| Extraction Data | Page 5 |
| Supplementary Figures and Tables |  |
| S1 - QuADS description | Page 7 |
| S2 - QuADS scoring | Page 8 |
| S3 - Summary of results | Pages 9-10 |

**Search Criteria**

Embase Classic+Embase

1 exp noise/ 169976

2 exp music/ 25492

3 exp illumination/ 47279

4 exp temperature/ 753713

5 exp thermodynamics/ 168456

6 noise.ti,ab. 205993

7 music.ti,ab. 29664

8 illumination.ti,ab. 47878

9 temperature.ti,ab. 835114

10 thermodynamics.ti,ab. 19906

11 1 or 2 or 3 or 4 or 5 or 6 or 7 or 8 or 9 or 10 1575550

12 exp surgeon/ 215945

13 exp surgery/ 6896731

14 exp anesthesist/ 45236

15 exp perioperative nursing/ 8116

16 exp operating room/ 60729

17 surgeon.ti,ab. 179432

18 surgical*.ti,ab. 1913986

19 anestheti*.ti,ab. 209057

20 anaestheti*.ti,ab. 103368

21 operating room.ti,ab. 51113

22 operating theatre.ti,ab. 6304

23 perioperative nursing.ti,ab. 1039

24 12 or 13 or 14 or 15 or 16 or 17 or 18 or 19 or 20 or 21 or 22 or 23 7660565

25 exp performance anxiety/ or exp task performance/ or exp job performance/ or exp mental performance/ or exp physical performance/ or exp performance/ or exp psychomotor performance/ or exp motor performance/ 1110284

26 exp medical error/ 203096

27 exp fatigue/ 360020

28 exp physiological stress/ 550608

29 25 or 26 or 27 or 28 1938527

30 11 and 24 and 29 6384

Ovid MEDLINE(R) <1946 to 2024>

1 exp Noise/ 28184

2 exp Music/ 17817

3 exp Lighting/ 13261

4 exp Temperature/ 465071

5 exp Thermodynamics/ 555184

6 noise.ti,ab. 127475

7 music.ti,ab. 18124

8 lighting.ti,ab. 9882

9 temperature.ti,ab. 489634

10 thermodynamics.ti,ab. 12128

11 1 or 2 or 3 or 4 or 5 or 6 or 7 or 8 or 9 or 10 997177

12 exp General Surgery/ 41260

13 exp Surgeons/ 19585

14 exp Anesthesia, Obstetrical/ or exp Anesthetists/ or exp Anesthesiology/ or exp Anesthesia/ or exp Anesthesia, General/ 231552

15 exp Perioperative Nursing/ 14082

16 exp Operating Rooms/ 16607

17 surgery.ti,ab. 1253481

18 surgeon.ti,ab. 100471

19 anaestheti*.ti,ab. 57746

20 anestheti*.ti,ab. 138904

21 anesthesiolog*.ti,ab. 33275

22 operating room.ti,ab. 29382

23 operating theatre.ti,ab. 3436

24 perioperative nurs*.ti,ab. 1837

25 12 or 13 or 14 or 15 or 16 or 17 or 18 or 19 or 20 or 21 or 22 or 23 or 24 1673797

26 exp Work Performance/ or exp Physical Functional Performance/ or exp Academic Performance/ or exp Psychomotor Performance/ 134684

27 exp Fatigue/ or exp Mental Fatigue/ 40532

28 exp Medical Errors/ 124622

29 exp Stress, Physiological/ or exp Stress, Psychological/ 443184

30 performance.ti,ab. 1054221

31 fatigue.ti,ab. 110886

32 26 or 27 or 28 or 29 or 30 or 31 1775866

33 11 and 25 and 32 1843

PubMed

("Noise"[mh] OR "Music"[mh] OR "Lighting"[mh] OR "Temperature"[mh] OR "Thermodynamics"[mh] OR noise[tiab] OR music[tiab] OR illumination[tiab] OR temperature[tiab] OR thermodynamics[tiab])

AND (surgeon[tiab] OR surgical*[tiab] OR anestheti*[tiab] OR anaestheti*[tiab] OR "operating room"[tiab] OR "operating theatre"[tiab] OR "operating room team"[tiab] OR "surgical team"[tiab] OR "perioperative team"[tiab] OR "theatre staff"[tiab] OR "scrub nurse*"[tiab] OR "circulating nurse*"[tiab]

AND ("Anxiety, Performance"[mh] OR "Task Performance and Analysis"[mh] OR "Job Performance"[mh] OR "Psychomotor Performance"[mh] OR "Motor Skills"[mh] OR "Medical Errors"[mh] OR "Fatigue"[mh] OR "Stress, Physiological"[mh] OR performance[tiab] OR error*[tiab] OR fatigue[tiab] OR stress[tiab]))

Cochrane CENTRAL

#1 (light*):ti,ab,kw

#2 MeSH descriptor: [Lighting] explode all trees

#3 (thermodynamic*):ti,ab,kw

#4 MeSH descriptor: [Thermodynamics] explode all trees

#5 (temperature):ti,ab,kw

#6 MeSH descriptor: [Temperature] explode all trees

#7 (music*):ti,ab,kw

#8 MeSH descriptor: [Music] explode all trees

#9 (noise):ti,ab,kw

#10 MeSH descriptor: [Noise] explode all trees

#11 #1 OR #2 OR #3 OR #4 OR #5 OR #6 OR #7 OR #8 OR #9 OR #10

#12 (surgeon):ti,ab,kw

#13 MeSH descriptor: [Surgeons] explode all trees

#14 (surger*):ti,ab,kw

#15 (anaesthe*):ti,ab,kw

#16 MeSH descriptor: [Anesthesia] explode all trees

#17 (anesthe*):ti,ab,kw

#18 MeSH descriptor: [Operating Rooms] explode all trees

#19 (operating room):ti,ab,kw

#20 #12 OR #13 OR #14 OR #15 OR #16 OR #17 OR #18 OR #19

#21 (performance):ti,ab,kw

#22 MeSH descriptor: [Performance Anxiety] explode all trees

#23 MeSH descriptor: [General Surgery] explode all trees

#24 MeSH descriptor: [Working Conditions] explode all trees

#25 MeSH descriptor: [Work Performance] explode all trees

#26 #21 OR #22 OR #23 OR #24 or #25

#27 #11 AND #20 AND #26

**Extraction Data

General information:**

- Title
- Name of journal / abstract / report that data are extracted from
- Lead author name
- Country in which the study conducted
- Year of publication
- Journal

## **Methods:**

- Aim of study
- Study design
- Was this a simulated study?
- Surgical specialty
- Was it a multi-centre study?

## **Participants:**

- Total number of senior staff (eg consultant, OR nurse) participants
- Total number of trainee participants
- Are there equal numbers of participants in the control and intervention groups?
- Duration of study ( eg days/weeks/months)

# **Intervention:**

- Environmental factor assessed
- How has the above factor been measured?
- Has the environmental factor in question been referenced or compared to defined standards?

# **Outcomes:**

- What is the primary outcome being measured? (eg impact of noise on ability to complete a surgical procedure)
- Is this an objective measurement or based on subjective staff opinion / survey?
- How many cases, or what time period, was this assessed over?
- Are there additional secondary outcomes listed?
- If this is a study about NOISE, what was the maximum and/or average decibel levels recorded? If this is a study about MUSIC, is there a stated % or number of people in the study who approved of music in the OR?
- What are the key findings relevant to the environmental factor(s) on surgical performance?
- Could you write a one-line summary of the key message?

**S1: QuADS**[(1)](https://paperpile.com/c/Ed8FRO/2zYK) *(abridged - examples in each section removed)*

| **Criteria** | **0** | **1** | **2** | **3** |
| --- | --- | --- | --- | --- |
| Theoretical/conceptual underpinning | No mention | General reference to broad theories | Identification of specific theories | Explicit discussion of theories with application throughout |
| Statement of aims | No mention | Referenced within report but no explicit statement | Aims statement but lacking detail or only in abstract | Explicit statement in main body of report |
| Clear description of target population | No mention | Description of area but not specific research environment | Setting included but lacking detail | Setting and target population explicitly discussed |
| Design appropriate for aims | No aims stated or totally unsuitable design | Can only address some aspect of the aims | Can address aims but there is more suitable alternative | Appears most suitable for the aims |
| Appropriate sampling for aims | No mention | Evidence of consideration | Evidence of consideration required for aims | Detailed evidence eg sample size calculation |
| Rationale for choice of data collection tools | No mention | Very limited explanation | Basic explanation of rationale | Detailed explanation inc relevance to aims |
| Format of tool(s) appropriate for aims | No research aims stated and/or data collection tool not detailed. | Suitable for some aspects of aims or to address them superficially | Can broadly address aims but could benefit from refinement | Allow for detailed data to be gathered around all relevant issues |
| Description of data collection procedure | No mention | Basic and brief outline | States each stage but with limited detail | Detailed description of each stage |
| Recruitment data provided | No mention | Minimal and basic | Some recruitment data, but incomplete | Complete and allows for full pic of recruitment outcomes |
| Justification for analysis method | No mention | Very limited | Basic justification | Detailed justification |
| Method of analysis appropriate for aims | No mention | Can only address aims basically/broadly | Can address aims but more suitable alternative exists | Most suitable for aims |
| Evidence research stakeholders considered | No mention | Basic consideration only | Evidence of stakeholder input informing the research | Substantial consultation identifiable in planning |
| Strength/limitations critically discussed | No mention | Very limited | Discussed but incomplete/lacking detail | Thorough discussion of all aspects |

**S2: QuADS Scoring**

| **Paper** | **QuADS Score** | **Paper** | **QuADS Score** |
| --- | --- | --- | --- |
| Arabaci (2021) | 24 | Narayanan (2024) | 20 |
| Brommelsiek (2022) | 23 | Narayanan (2023) | 21 |
| Byrne (2023) | 32 | Narayanan (2018) | 19 |
| Chandrasekar (2024) | 19 | Padmakumar (2016) | 18 |
| Cheriyan (2016) | 20 | Palejwala (2023) | 28 |
| Dholakia (2015) | 28 | Palejwala (2019) | 29 |
| Engelmann (2014) | 33 | Palejwala (2024) | 19 |
| George (2011) | 20 | Palejwala (2025) | 32 |
| Gülsen (2021) | 24 | Peisl (2024) | 32 |
| Han (2022) | 30 | Shover (2021) | 34 |
| Hemphälä (2020) | 34 | Rubbi (2024) | 31 |
| Hogan (2015) | 19 | Srivastava (2021) | 16 |
| Idrees (2024) | 21 | Stadler (2023) | 25 |
| Kurmann (2011) | 23 | Tseng (2022) | 21 |
| Lehrke (2022) | 19 | Tsiou (2008) | 19 |
| Makama (2010) | 12 | Ukegjini (2020) | 30 |
| Morghen (2009) | 19 | Ward (2021) | 32 |

**S3: One-line summary of paper**

| Arabaci (2021) | Mean intraoperative noise levels were higher than recommended limits, and increased noise correlates with increased staff anxiety. |
| --- | --- |
| Brommelsiek (2022) | Human caused sounds can disproportionately interfere with team communication and performance |
| Byrne (2023) | Thermal discomfort during surgery can be reduced and surgeon comfort increased, but there is no discernible benefit on cognitive function. |
| Chandrasekar (2024) | Although music subjectively improved surgeon stress, there was no significant improvement in performance/cognition |
| Cheriyan (2016) | Noise pollution decreases effective communication during PCNL |
| Dholakia (2015) | Higher noise levels in theatre may contribute to an increase in SSIs, due to the effect on the operation |
| Engelmann (2014) | Implementing a noise-reduction programme can significantly reduce postop complications, although the response varies between individual surgeons (or 'responders') |
| George (2011) | If played at appropriate volumes, the majority of staff find music played in the OR is beneficial for their overall performance |
| Gülsen (2021) | Ambient noise in the OR is above recommended safe levels, and the majority of staff working there have experienced negative physiological and psychological effects due to this. |
| Han (2022) | Laparoscopic teams performed better with music than in a noisy environment, and surgeons with more experience were less affected by a noisy environment. |
| Hemphälä (2020) | Better general lighting may be an efficient way to improve surgical results and medical safety. |
| Hogan (2015) | Implementing a successful noise reduction programme is feasible with staff education, and this could lead to a less stressful and safer working environment |
| Idrees (2024) | Both subjective and novel objective data (salivary cortisol) shows that noise exposure causes an increased stress response in surgeons, which has implications for performance and safety. |
| Kurmann (2011) | Increased noise levels in theatre may be associated with an increased risk of postoperative wound infection |
| Lehrke (2022) | A headphone-based noise reduction system may reduce stress in operating theatre staff during cardiac and urologic surgery |
| Makama (2010) | Surveyed theatre staff in a Nigerian hospital perceived music in theatre to be beneficial for staff and patients |
| Morghen (2009) | Changes in the illumination did not have statistically significant effects on staff stress/performance |
| Narayanan (2024) | Surgeons broadly perceive music in the OR as a positive way to reduce stress and anxiety, but this needs to be agreed by the team to reduce the potential for conflict. |
| Narayanan (2023) | Music is used widely and is viewed by this cohort of surgeons as improving things like mood and team performance, but concerns regarding the effect on distractedness and communication remain. |
| Narayanan (2018) | Music is seen as improving calmness, mood, surgeons’ and overall team performance, but can impair communication and is viewed negatively in the context of critical theatre situations and urgent procedures. |
| Padmakumar (2016) | Multi-disciplinary staff feel noise adversely affects multiple human factor domains in the operating theatre. Music is generally seen as positive but needs further research. |
| Palejwala (2023) | Heat exposure can increase mental workload, but staff can maintain cognitive function and manual dexterity even in high temperatures. |
| Palejwala (2019) | There was no deterioration in performance in hotter ambient temperatures, although it was concerning that many staff were objectively dehydrated. |
| Palejwala (2025) | Psychological stress in combination with heat stress, but perhaps not those stressors alone, can negatively impact cognitive speed and working memory and increase perceived workload. Heat strain, caused by a hot OT, can be exacerbated by psychological stress |
| Palejwala (2024) | The operating temperature in burns surgery varies significantly by country and there is no standard consensus. |
| Peisl (2024) | Noise in theatre does not independently predict postoperative complications when surgical difficulty is taken into account. Noise in theatre is consequence of surgical difficulty, rather than a cause of postoperative complications. |
| Rubbi (2024) | There is a non-significant trend towards more positive emotions and reduced stress in healthcare professionals who use music in the OR, compared to those who don't. |
| Shover (2021) | music had no effect (either positive or negative) on the performance of novice surgeons performing a simulated, complex surgical task |
| Srivastava (2021) | Background ambient noise is felt to negatively affect communication and stress by the majority of OR staff, but this does not extend to music. |
| Stadler (2023) | Industry-grade noise cancelling headphones are not beneficial in reducing distracting noise intra-operatively. |
| Tseng (2022) | Increasing sound levels (even to 75-80db) or the addition of music did not affect situational awareness results for OR nursing staff. |
| Tsiou (2008) | Noise in Greek operating theatres regularly exceeded safe limits, and a majority of staff felt it negatively impacted on their job. |
| Ukegjini (2020) | A noise warning device in the OR can decrease overall ambient noise levels and surgeon stress, but has no sig benefit on postop morbidity. |
| Ward (2021) | Heat exposure affects the cognitive performance of the entire operative team, both objectively and subjectively, and this is magnified as the time period increases. |

**References**

1. [Harrison R, Jones B, Gardner P, Lawton R. Quality assessment with diverse studies (QuADS): an appraisal tool for methodological and reporting quality in systematic reviews of mixed- or multi-method studies. BMC Health Serv Res. 2021 Feb 15;21(1):144.](http://paperpile.com/b/Ed8FRO/2zYK)
